# Multiplex PCR technique could be an alternative approach for early detection of leprosy among close contacts - a pilot study from India

**DOI:** 10.1186/1471-2334-10-252

**Published:** 2010-08-24

**Authors:** Surajita Banerjee, Kamalesh Sarkar, Soma Gupta, Prasanta Sinha Mahapatra, Siddhartha Gupta, Samudra Guha, Debasis Bandhopadhayay, Chaitry Ghosal, Suman Kalyan Paine, Rathindra Nath Dutta, Nibir Biswas, Basudev Bhattacharya

**Affiliations:** 1 Department Of Biochemistry, IPGME&R, Kolkata, India; 2 National Institute Of Cholera and Enteric Diseases, Kolkata, India; 3 Department Of Biochemistry, N.R.S. Medical College, Kolkata, India; 4 Department of Dermatology, IPGME&R, Kolkata, India; 5 Department of Dermatology, Calcutta Medical College, Kolkata, India

## Abstract

**Background:**

Implementation of Multi drug Therapy (MDT) regimen has resulted in the decline of the total number of leprosy cases in the world. Though the prevalence rate has been declining, the incidence rate remains more or less constant and high in South East Asian countries particularly in India, Nepal, Bangladesh, Pakistan and Srilanka. Leprosy, particularly that of multibacillary type spreads silently before it is clinically detected. An early detection and treatment would help to prevent transmission in the community. Multiplex PCR (M-PCR) technique appears to be promising towards early detection among contacts of leprosy cases.

**Methods:**

A total of 234 paucibacillary (PB) and 205 multibacillary (MB) leprosy cases were studied in a community of an endemic area of Bankura district of West Bengal (Eastern India). They were assessed by smear examination for acid-fast bacilli (AFB) and M-PCR technique. These patients were treated with Multidrug Therapy (MDT) as prescribed by WHO following detection. A total of 110 MB and 72 PB contacts were studied by performing M-PCR in their nasal swab samples.

**Results:**

83.4% of MB patients were observed to be positive by smear examination for AFB and 89.2% by M-PCR. While 22.2% of PB patients were found to be positive by smear examination for AFB, 80.3% of these patients were positive by M-PCR. Among leprosy contacts (using M-PCR), 10.9% were found to be positive among MB contacts and 1.3% among PB contacts. Interestingly, two contacts of M-PCR positive MB cases developed leprosy during the period of two years follow up.

**Conclusion:**

The M-PCR technique appears to be an efficient tool for early detection of leprosy cases in community based contact tracing amongst close associates of PB and MB cases. Early contact tracing using a molecular biology tool can be of great help in curbing the incidence of leprosy further.

## Background

From time immemorial, leprosy is a grossly mutilating disease associated with social stigma and taboos, particularly in underdeveloped nations. The global caseload of leprosy has reduced by almost 90% over the last 20 years and 15 million cases have been detected and cured worldwide. Three hundred thousand (0.3 million) new cases were detected during the year 2005 [1]. The principal factor contributing to this worldwide success is attributed to the introduction of standardized MDT regimens against the causative agent, *Mycobacterium leprae*. Further, leprosy elimination campaigns for case detection in communities, training of physicians and leprosy health care workers, community awareness towards prevention and control of leprosy have also proven to be beneficial. **Elimination is defined as a reduction in the prevalence of leprosy patients receiving antibacterial therapy to less than 1 per 10,000 populations**[2], which indicates that the disease is no longer considered a major public health problem. India has achieved the prevalence rate of less than 1 per 10,000 populations in 2006. But the incidence rate remains high in six countries of South East Asian region including India. India alone accounted for 60% of the world's newly detected cases [1,3]. This might be due to lack of consistent information on the core elements of this infectious disease, e.g. source of infection, reservoir and mode of transmission, host factors related to immunity of disease etc [4-6]. It has been observed that though prevalence has declined since initiation of MDT, the incidence has not shown a similar decline during the same period i.e. after implementation of MDT [7-10].

Leprosy, particularly MB type, is highly contagious and infectious may spread to several contacts of patients even before clinical diagnosis. In case of MB leprosy, contacts staying in the same house are at higher risk of getting infection compared to contacts staying at the neighboring houses [10-14]. Therefore, early detection of infections among close contacts followed by effective chemotherapy is likely to bring down the spread of disease leading to a decline in the overall incidence rate. Unfortunately, the conventional method of contact tracing fails to detect fresh cases before it becomes transmissible to others (person to person transmission) [15] Hence, it is necessary to have an alternative and more effective tool for an early detection to prevent and control further transmission.

Previous work carried out by us, has led to the development of a Multiplex PCR (M-PCR) for early diagnosis of leprosy. The technique was standardized and was evaluated with high sensitivity and specificity [16].

The present study was carried out to evaluate whether the same technique could be used as a better diagnostic tool for early detection of leprosy cases and contacts for prediction of future cases of leprosy.

## Methods

### Patients

This study was conducted in an endemic population in the district of Bankura (prevalence rate greater than 2 per 10,000 populations) in West Bengal, India. After taking formal consent, a total no of 234 paucibacillary (PB) and 205 multibacillary cases (MB) attending the Public Health Centers were assessed by AFB (Acid Fast Bacilli) smear examination as well as multiplex-PCR (M-PCR) to assess the diagnostic efficiency of the later. Out of 234 PB cases 140 were tuberculoid (TT) and 94 were borderline tuberculoid (BT). Similarly, of 205 MB cases 53 were borderline lepromatous (BL) and 152 were lepromatous leprosy (LL)

Patients were grouped in the following categories:

(i) Patients without treatment, (ii) Patients on treatment not more than two months, (iii) Patients complaining of hypoesthesia but showing no clinical symptoms of leprosy - considered as Indeterminate type, and (iv) Patients released from treatment (RFT) and later developed a new active lesion/i.e. relapsed cases.

Slit skin smear (SSS) for acid -fast bacilli (AFB) staining were obtained from all patients for determination of Bacterial Index (BI). All diagnosed cases were given MDT as per the national leprosy control programmed guidelines [17]. Competent health care workers followed up household contacts of these patients. A total of 182 persons of which 110 were MB contacts and 72 were PB contacts, participated in this study voluntarily. Nasal swabs/slit skin smear specimens were obtained from all contacts after obtaining their necessary consent. The contacts were followed up for two years for observing the development of clinical leprosy.

**Ethical approval **was taken from the Ethical Committee of the Institute (Office of the Director, Instt of Post Graduate Medical Education & Research, Kolkata, Govt. Of West Bengal). Ref No. Inst/IEC/1835 dated 2.8.05 as a part of the project entitled "Development of Multiplex PCR for Early Diagnosis and Strain Differentiation of *M.leprae*" and since then reviewed periodically.

### Sample collection

Slit Skin Smear: SSS were obtained from each patient (from 3 to 6 sites, depending on the type of leprosy) for determination of bacterial index (BI). 4 mm punch biopsy/SSS from three to six sites for each patient were obtained. Half of the biopsy samples from each patient was used for paraffin embedded sectioning and the other portions were stored at -20°C until PCR was performed. BI (bacteriological index) was also determined microscopically from paraffin section of biopsy specimens.

Collection of Nasal Swabs: The surface of the nasal septum either side of each patient were swabbed with sterilized wet cotton swabs, frozen in buffered saline containing 0.05% Tween80, which released the sample from cotton swabs. The aliquot was centrifuged at 10,000X g. The sediment was processed for DNA extraction as described bellow.

### DNA Extraction from clinical samples

#### Extraction of DNA

a) From Skin Tissues: The Frozen section of tissues/skin biopsy specimens were cut to small pieces with sterile scissors. These samples were homogenized in a manual homogenizer with 1 ml sterile distilled water. It was then incubated in lyses buffer containing 300 μl of 100 mM Tris-HCl, pH8.5 (containing 0.05% Tween 20 and 60 μg of proteinase K per ml) for 18hrs at 60°C. Paraffin oil (40 μl) was layered on top of the buffer to prevent evaporation. Thereafter, the samples were incubated at 97°C for 15 minutes [11], followed by heating for inactivation of proteinase K. Equal volume of phenol-chloroform-isoamyl alcohol (25:24:1) was later layered on the lysed homogenated product. The tube was shaken vigorously for 1 minute. After centrifugation of the material for 8 minutes, the aqueous phase was collected and again mixed with an equal volume of chloroform-isoamyl alcohol. This was followed up by another brief centrifugation. Then, the uppermost phase was collected and boiled for 10 minutes to destroy DNase, followed by precipitation of DNA with ethanol. The precipitated DNA was resuspended in 100 μl of distilled water and used for M-PCR.

b)Extraction of DNA from Nasal swab: Frozen samples were quickly thawed and centrifuged at 10,000X g for 20 minutes The sediment was subjected to DNA extraction following the same procedure as mentioned above.

### Multiplex PCR

A M-PCR was developed in our laboratory [16] based on:

(a) Primers amplifying the 372 bp of the repetitive sequence of *M.leprae*, known to be specific for *M.leprae *and is not present in twenty other mycobacterial species [19].

(b) A pair of primers was designed to amplify 201 bp flanking entire21 TTC repeats [20].

Sequences for (a): 5'-CGG CCG GAT CCT CGA TGC AC-3' (primerR1)

5'-GCA CGT AAG CTT GTC GGT GG-3' (primerR2)

For (b): 5'-GGA CCT AAA CCA TCC CGT TT-3' (TTC-A)

5'-CTA CAG GGG GCA CTT AGC TC-3' (TTC-B)

Reaction mixtures, conditions of reactions and cycling conditions were optimized as follows:

The reaction mixture contains 50 ul of 10 mMTris-HCL(pH 8.3), 50 mMKCL, 1.5 MgCl2, 0.01% (wt/Vol) gelatin, 200 uM each dATP, dGTP, dCTPand dTTP, 1U of Taq polymerase(Perkin -Elmer Cetus, Norwalk, Conn)0.5 μm each primer and DNA extracted from biopsy samples.

PCR Condition: PCR is carried out in a thermocycler for 35 cycles consisting of denaturation at 94°C for 1 min, annealing at 60°C for 2 mins and primer extension at 72°C for 3 mins. After purified DNA is added to the PCR mix, triple distilled water is used as negative control. The tubes are kept for at least 10 mins at room temperature. After amplification is finished, a 20 μl portion of the reaction mixture is run in a 2% agarose gel. After electrophoresis, the gel is stained with ethidium bromide, and the 372 bp and 201 bp bands examined under UV illumination.

### Statistical Analysis

Sensitivity of smear examination for AFB and M-PCR of skin biopsy samples is calculated considering clinically diagnosed cases as true gold standard of positivity.

## Results

The findings of M-PCR and BI of SSS are presented in table 1.

**Table 1:** Sensitivity of AFB and multiplex-PCR as diagnostic tools in detecting paucibacillary as well as multibacillary leprosy cases among clinically diagnosed patients

Type of disease	Clinical Forms	Cases confirmed by AFB In SSS (Slit skin smear)	Cases confirmed by Multiplex-PCR	Percent of positivity by AFB test	Percent of positivity of M-PCR test
Paucibacillary cases (n = 234)	TT (n = 140)	44 (BI < 2)	111	31.4%	79.2%
	
	BT (n = 94)	18 (BI < 2)	77	19.1%	81.9%

Total	234	52	188	22.2%	80.3%

Multibacillary cases (n = 205)	BL (n = 53)	38 (BI > 2)	47	71.6%	88.6%
	
	LL (n = 152)	133 (BI > 2)	136	87.5%	89.4%

Total	205	171	183	83.4%	89.2%

**Figure 1:**
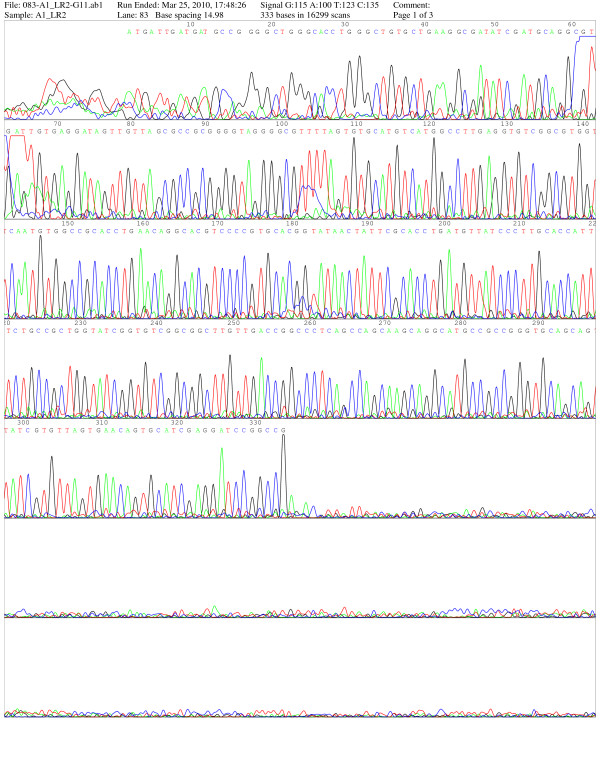
**Sequencing report**.

From the above table it is clear, that on the whole M-PCR can detect a higher number (79.2% to 89.4%) of leprosy cases compared to SSS test (19.1% to 87.5%). The sensitivity of M-PCR in case of tuberculoid type is higher than smear examination. It is noted that the positivity was considerably higher in tuberculoid cases (TT 79%, BT 81.9%) in comparison to SSS (TT 31.4%, BT 19.1%). Multiplex PCR of nasal swab was found to be positive 49.4% (42/85), 51.6% (32/62), 62.5% (25/40) and 78.1% (75/96) in the categories of TT, BT, BL and LL respectively. This indicates that nasal swab could be an alternative, non-invasive procedure for M- PCR for leprosy detection. PCR results were confirmed by sequencing. (Figure -1)

The data obtained from M-PCR of nasal swab of 110 household contacts of MB patients and 72 of PB patients are presented in table 2. The result shows that 11.7% adults and 9.5% children among MB contacts and 2% adult among PB contacts showed evidence of *M leprae *DNA in their nasal swabs. These contacts were followed up prospectively for two years since the day of collection of nasal swabs. It was observed that 1 adult and 1 child developed leprosy from M-PCR positive MB contacts during the two-year observation period. None of the contacts from PB cases developed clinical leprosy during the follow up period (table-2)

**Table 2:** Showing M-PCR positivity and incidence of leprosy in household contacts of leprosy cases in nasal swabs

Multibacillary leprosy contact			Paucibacillary leprosy contact		
**Number of persons tested with M-PCR** **(Nasal Swab)**	**Number positive (%)**	**Development of leprosy cases among contacts within 2 year follow up**	**Number of persons tested with M-PCR (Nasal Swab)**	**Number positive (%)**	**Development of leprosy cases among contacts within 2 year follow up**

Adult = 68	8 (11.7%)	1	Adult = 48	1 (2%)	0

Child = 42	4 (9.5%)	1	Child = 24	0 (0%)	0

Total = 110	12 (10.9%)	2	Total = 72	1 (1.3%)	0

## Discussion

Leprosy is a disease with long incubation period and the symptoms are difficult to perceive at the early stage of infection. Self-healing does occur in a large number of infected cases. Moreover many patients with early signs are not aware that they are suffering from leprosy and thus clinical diagnosis is often delayed. Clinical diagnosis is possible only when the patient is symptomatic, exhibiting lesions and satisfies the criteria of cardinal signs of leprosy [23]. A sizable proportion of new cases are among children (WHO 2004),[21] as they often remain in close contact with infected family members sharing same dwelling units that facilitate infection in them [22] Similarly, an infected child could pass this infection to other children while they are in contact for longer duration, i.e, playing in a group or in school. This often goes un-noticed during initial phase because of its slow and silent nature of early transmission.

Considering the above, early detection of cases followed by effective chemotherapy appears to be the single most effective strategy for reducing incidence of leprosy cases as well as to prevent transmission. However, existing method of contact tracing and detection is not beyond criticism. The results of M-PCR that was developed and used in this pilot study as an alternative tool appears to be encouraging. In our study using M-PCR, we found that the molecular tool is much better in detecting high percentage of TT/BT cases (79.2% & 81.9% respectively), than cases detected by SSS (22.2%). The overall positivity of PB cases by PCR is 80.3%. As the disease progresses further, the positivity of both PCR (80.3% to89.2%) and AFB smear examination are (22.2%to83.4%) increased. This efficacy of M-PCR over SSS can be explained by the fact that, with the progression of disease there is increase of bacterial load resulting in availability of more genomic DNA of *M leprae *and thereby leading to PCR positivity. While specific PCR for diagnosis of leprosy developed by previous scientists [27,28] were successful in diagnosing only 50% of PB cases, the present M-PCR was able to diagnose 80.34% of PB cases. The reason for identifying more cases of early infection might be due to the use of combination of two specific primers in the same PCR reaction. However, unlike the previous workers, the M-PCR failed to detect 100% cases of MB leprosy. The reason for the failure may be due to the inclusion of large number of cases (205) compared to those of 37[28] and 38 [27] cases of MB leprosy. From table 3 and 4 it is seen that MPCR is highly sensitive in new cases and the sensitivity gradually decreases in case of RFT/Relapse cases both in PB and MB patients. This result is along expected lines, as it is difficult to amplify mycobacterial DNA in patients after treatment due to genomic degradation of the bacteria. The specificity was not calculated in this study as the same was done in our earlier study while evaluating MPCR as a diagnostic tool and it was found to be very high [16]. From the present study, it can be suggested that M-PCR could be an alternative screening tool for detection of early leprosy cases or their close contacts with high sensitivity compared to any other available tools.

**Table 3:** Sensitivity of AFB and multiplex-PCR as diagnostic tools in detecting multibacillary leprosy cases among different categories of multibacillary patients

BL n = 53					
Clinical status of the disease	Cases confirmed by AFB In SSS(Slit skin smear)		Cases confirmed by Multiplex-PCR	Percent of positivity by AFB test	Percent of positivity of M-PCR test
	BI	No of case			
Patients without treatment (New Cases)(n = 44)	2	23	23	100%	100%
	3	11	11		
	4	10	10		
Patients on treatment not more than 2 months (n = 5)	0	4	2(50%)	25%	60%
	2	1	1		
	3	0	0		
	4	0	0		
Patients released from treatment (RFT)^(a)^/later developed a new active lesion/relapsed cases^(b) ^(n = 4)	0	2^a,^	2(0%)	50%	50%
	2	1^a^	1^a^		
	3	1^b^	1^b^		
Total n = 53				71.6% (38/53)	88.67% (47/53)
LL n = 152					
**Clinical status of the disease**	**Cases confirmed by AFB In SSS(Slit skin smear)**		**Cases confirmed by Multiplex-PCR**	**Percent of positivity by AFB test**	**Percent of positivity of M-PCR test**
	**BI**	**No of case**			
Patients without treatment (New Cases)(n = 115)	2	8	8	100%	100%
	3	35	35		
	4	37	37		
	5	31	31		
	6	4	4		
Patients on treatment not more than 2 months (n = 12)	0	4	1(25%)	66.66%	75%
	2	3	3		
	3	2	2		
	4	3	3		
	5	0	0		
	6	0	0		
Patients released from treatment (RFT)^(a)^/later developed a new active lesion/relapsed cases^(b) ^(n = 25)	0	14^a^,1^b^	2(13.33%)	40%	48%
	2	1^a^,2^b^	3		
	3	2^b^	2		
	4	2^a^,1^b^	3		
	5	1^a^	1		
	6	1^a^	1		
Total n = 152				83.4% (133/152)	89.2% (136/152)

**Table 4:** Sensitivity of AFB and multiplex-PCR as diagnostic tools in detecting paucibacillary as well as multibacillary leprosy cases among different categories of paucibacillary patients

Clinical status of the disease	Cases confirmed by AFB In SSS(Slit skin smear)		Cases confirmed by Multiplex-PCR	Percent of positivity by AFB test	Percent of positivity of M-PCR test
	BI	No of case			
Patients without treatment (New Cases)(n = 128)	0	85	62 (72.9%)	50.58%	82%
	1	43	43		
Patients on treatment not more than 2 months (n = 7)	0	6	3 (50%)	14.28%	57.14%
	1	1	1		
Patients released from treatment (RFT)^(a)^/later developed a new active lesion/relapsed cases^(b) ^(n = 3)	0	3^a^	1^a ^(33.3%)	0%	33.33%
	1	0	0		
Patients complaining of hypoesthesia but showing no clinical symptoms of leprosy - considered as Indeterminate type(n = 2)	0	2	1 (50%)	0%	50%
	1	0	0		
Total n = 140				31.4% (44/140)	79.2% (111/140)
BT n = 94					
**Clinical status of the disease**	**Cases confirmed by AFB In SSS(Slit skin smear)**		**Cases confirmed by Multiplex-PCR**	**Percent of positivity by AFB test**	**Percent of positivity of M-PCR test**
	**BI**	**No of case**			
Patients without treatment (New Cases)(n = 85)	0	68	55 (80.8%)	20%	84.70%
	1	17	17		
Patients on treatment not more than 2 months (n = 6)	0	5	2(40%)	16.6%	50%
	1	1	1		
Patients released from treatment (RFT)^(a)^/later developed a new active lesion/relapsed cases^(b) ^(n = 3)	0	2^a^, 1^b^	1^a^,1^b ^(33.33%)	0%	66.6%
	1	0	0		
Total n = 94				19.14% (18/94)	81.9% (77/94)

TT (including 2 indeterminates) n = 138 + 2 Indeterminate

Another important and effective strategy for reduction of incidence of leprosy is contact tracing and early detection of cases among them. In our pilot study M-PCR technique was applied in the leprosy contacts for adopting a better way of community-based early case detection. Contacts of PB and MB cases both were followed up after testing their nasal swabs by M-PCR. It was noted that two of the twelve M-PCR positive cases developed as clinical cases and none of the M- PCR negative cases developed any form of leprosy during this period. It is interesting to note that a significantly larger numbers (12 numbers) of MB contacts were positive by M-PCR than that of PB contacts (2 numbers) who did not develop leprosy during the follow up period.

It has been estimated that 6-8% of household contacts develop clinical symptoms of leprosy within two years of follow up since the diagnosis of the index cases[24]. In our study 10.9% MB contacts were PCR positive and 1.8% developed the disease within two years of study period. This low number of detection in our study group could be due to the lower transmission dynamics in the community during the post elimination era. Keeping in mind the long incubation period of the disease it is imperative that the contacts need to be followed and observed for a longer period.

The trend that we find in our study can serve as important clue that the contacts of MB patients are at increased risk of developing leprosy in the future. This hypothesis needs to be tested on large scale of population over a long interval before coming to any conclusion, since the authors have no intention to put forth the MB patients into social ostracism under the fear of infection. Whatever the relationship between positivity of PCR and development of the disease, PCR is much more sensitive than microscopic examination for direct detection of bacilli [25,26]. Moreover, at present there is no other more sensitive alternative tool available for early detection of leprosy other than PCR and serological test [22]. Present M-PCR test has been standardized based on the presence of *M. leprae *DNA which does not support the presence of viable organisms. Effort should be made to standardize a method based on mRNA detection so that viability of *M. leprae *bacilli could also be ascertained [27-30].

It should be also noted that the PCR positivity in contact persons indicate the presence of *M. leprae *DNA only and not infection and therefore, a careful follow up of them should be done and treatment should be started immediately after the development of first sign and symptoms of leprosy.

## Conclusion

This study indicates that M-PCR can be used as an efficient tool for early detection of leprosy among contacts. However it needs further in-depth study with adequate population size and controls over a long period of time. As this M- PCR can only detect the presence of *M. leprae *DNA, hence a contact with positive PCR result must be followed up regularly for detection of any development of disease. As leprosy has a long and varied incubation period, a long term follow up/observation is necessary to establish clearly the early case detection efficiency of M- PCR.

This M- PCR is a relatively expensive procedure compared to other methods of detection of infection caused by *M. leprae*. It has its limitations in the viability detection of the bacteria. In addition, it also requires a well-equipped laboratory which is difficult to replicate in the field. When tests are performed in bulk, the cost is expected to go down significantly. Hence, we recommend its application in a large number of samples to make it cost effective. The test needs to be evaluated further as it could serve as a better diagnostic tool for early case detection and their treatment to achieve faster control of leprosy.

## Competing interests

The authors declare that they have no competing interests.

## Authors' contributions

SB: Developed and standardized the protocol, wrote the manuscript. PSM & SG: Collected the samples. SG, SG: Wrote and edited the manuscript, DB, CG, SKP, RND, NB: edited the manuscript. KS: Did the statistical analysis. BB: Guided the overall work and helped in writing and editing the manuscript. All the authors read and approved the manuscript.

## Pre-publication history

The pre-publication history for this paper can be accessed here:

http://www.biomedcentral.com/1471-2334/10/252/prepub
